# Health workers’ attitudes toward immigrant patients: a cross-sectional survey in primary health care services

**DOI:** 10.1186/1478-4491-10-14

**Published:** 2012-07-09

**Authors:** Sónia Dias, Ana Gama, Helena Cargaleiro, Maria O Martins

**Affiliations:** 1 Instituto de Higiene e Medicina Tropical & CMDT, Universidade Nova de Lisboa, Rua da Junqueira 100, 1349-008, Lisbon, Portugal; 2 Ministry of Health, Avenida João Crisóstomo 14, 1000-179, Lisbon, Portugal; 3 Instituto de Higiene e Medicina Tropical & UPMM, Universidade Nova de Lisboa, Rua da Junqueira 100, 1349-008, Lisbon, Portugal

## Abstract

**Background:**

Health workers’ attitudes toward immigrant patients influence behaviour, medical decisions, quality of care and health outcomes. Despite the increasing number of immigrant patients in health services and the potential influence of health workers’ attitudes, there is little research in this area. This study aimed to examine attitudes of different health workers’ groups toward immigrant patients and to identify the associated factors.

**Methods:**

This cross-sectional study was conducted with a random sample of 400 health workers from primary health care services in the Lisbon region, Portugal. Among those, 320 completed a structured questionnaire. Descriptive analysis and multiple linear regression analysis were used for the evaluation of data.

**Results:**

Most participants did not agree that immigrant patients tend to behave like victims, but about half considered that some are aggressive and dangerous. Doctors and nurses showed more positive attitudes than office workers. Among doctors, the older ones reported less positive attitudes compared to the younger ones. Health workers who have less daily contact with immigrants revealed more positive attitudes. Most participants evaluated their knowledge and competencies to work with immigrants as moderate or low.

**Conclusions:**

Although health workers reveal positive attitudes, this study reinforces the need to develop strategies that prevent negative attitudes and stereotyping in health services. Efforts should be made to improve workers’ competencies to deal with culturally diverse populations, in order to promote quality of health care and obtain positive health outcomes among immigrant populations.

## Background

Migratory flows have resulted in a growing cultural heterogeneity within the population in Europe. In Portugal, the resident foreign born population represented 6.1% of the total population in 2008 and mostly came from Brazil, Portuguese-speaking African countries and Eastern European countries [1,2].

It has been acknowledged that including the immigrant population in the core institutions of host societies, namely the health care system, is crucial for positive integration [3,4]. Indeed, national efforts have been made in the last decades to improve access and utilization of health services by immigrants in Portugal. Currently, all citizens have the right to be attended in the National Health Service (NHS) for primary and secondary health care, according to their need and irrespective of their nationality, economic means or legal status. Health care is free of charge for children under 12 years of age, pregnant women and recent mothers, patients in family planning programs, unemployed persons registered at a job centre and their dependants, recipients of welfare benefits and individuals with legally recognised chronic diseases. Those without a residency or work visa or who do not pay social security must pay the full fare for services.

In the context of cultural diversity, how to improve delivery and quality of health care to culturally diverse populations has been a major concern. Recent research has provided evidence that immigrants frequently underuse health services and tend to receive a lower quality health care than the general population [5-7].

Disparities in the quality of health care can result from a combination of factors at the patient, health worker and services levels [6-8]. In particular, the professional-patient relationship has been documented as playing an important role in patient care [9-11]. The interaction between health workers and immigrants is affected by socio-demographic and cultural background, expectations of health care, language barriers and communication difficulties [10,12]. Additionally, studies indicate that the professional-patient relationship may also be mediated by health workers’ own beliefs, perceptions and attitudes toward immigrants [11,13,14]. Attitudes toward immigrants and negative stereotypes have been associated with health workers’ characteristics, professional experience and lack of knowledge and competencies to deal with cultural diversity [13,15,16].

Health workers’ stereotyping and prejudice toward immigrants influence behaviour when interacting with these patients and making medical decisions, namely with regard to diagnosis and treatment prescription [6,7,17,18]. Patients themselves perceive these attitudes and their reaction may be reflected in low satisfaction, non-compliance with treatment and reduced utilization of services, which negatively affects care outcomes [10,15,18,19].

Despite the potential influence of health workers’ attitudes toward immigrants, there is a lack of investigation in this area. Research has mainly consisted of qualitative studies, with a limited number of participants, while quantitative surveys are scarce [12,20]. Moreover, several investigations have focused on the patient side of the professional-patient relationship, while the perceptions and attitudes of health workers have not yet been well explored [8,12].

This study aimed to examine the attitudes of different health workers’ groups toward immigrant patients and associated factors. The study also provides information about health workers’ self-perception on their knowledge and competencies to work with immigrant patients.

## Methods

### Sample and data collection

This study was based on a cross-sectional survey conducted between January and April 2009. The study sample consisted of health workers from primary health care services in the Lisbon region (which has the highest concentration of immigrants in the country). Of the 80 primary health care services in the region, 40 were randomly selected; then, in each service, 10 health workers were randomly selected. Among the sample of 400 health workers, 320 (80%) agreed to participate in the study: 115 office workers (36.0%), 114 nurses (35.6%) and 91 doctors (28.4%).

Data were collected using a self-administered questionnaire. The questionnaire was pilot-tested for validity among a group of health workers not involved in the study. This allowed us to ensure the readability and clarity of the questions and to revise the instrument for final use. The questionnaire included closed questions on demographic characteristics and professional experience, knowledge and competencies to work with immigrant patients and attitudes toward this group.

Participation of health workers in the study was voluntary, anonymous and confidential.

The study was approved by the Ethical Committee of the Institute of Hygiene and Tropical Medicine, New University of Lisbon.

### Variables

Demographic characteristics of participants included gender and age. Professional experience was based on the frequency of contact with immigrant patients and was measured by the number of immigrant patients contacted daily; the response options were *‘*less than three’, *‘*four to seven’ and ‘eight or more’. Respondents were asked to report how they perceived their knowledge and competencies to work with immigrant patients with three response options of ‘low’, ‘moderate’ and ‘high’. Participants also reported their opinion about the importance of receiving specific training about immigrants’ health for improving professional performance, with response options being ‘not important’, ‘indifferent’ and ‘important’.

To assess health workers’ attitudes toward immigrant patients, participants reported to what extent they agreed with the following six statements: Some immigrants are aggressive and dangerous; Immigrants take advantage of the social benefits that Portugal offers them; Immigrants are more claiming than the general population; Immigrants often dramatise and exaggerate their problems; Frequently immigrants do not respect the health services' rules; When immigrants come to my service they behave like victims. Responses for each item had five options ranging from ‘strongly agree’ (1) to ‘strongly disagree’ (5).

### Statistical analysis

The associations between demographic characteristics, frequency of contact with immigrant patients, self-perception on knowledge/competencies, attitudes toward immigrant patients and the three health workers’ groups were analyzed using the Chi-square test (for categorical variables) and the Kruskal-Wallis test (for age). For this analysis, items related to attitudes were aggregated in three categories: ‘strongly disagree and disagree’; ‘neither agree nor disagree’; ‘strongly agree and agree’).

A multiple linear regression analysis was used to estimate the relationship between the variables gender, age, health workers’ group, frequency of contact with immigrant patients, and the variable ‘attitudes toward immigrant patients’. This variable results from the sum of the responses of each participant to the six items related to attitudes, and ranges from 1 to 25 (items were recoded so that higher scores reflected more positive attitudes). The original items were found to have adequate reliability (Cronbach's alpha = 0.78).

In the first model, age was included as a continuous variable; however, no significant effect was found. We examined the interaction of age x health workers’ group. In a second model the significant interaction term for age and doctors was included. The sign and the significance of the interaction term reveal the extent to which there are differences between younger and older doctors regarding attitudes toward immigrant patients.

All analyses were performed using PASW 18 software.

## Results

### Health workers’ demographic and professional experience characteristics

Of the total sample, 84.1% of the participants were women; the proportion of women was higher among office workers (93.9%) and nurses (92.1%) than among doctors (61.5%) (*P* <0.001) (Table 1). The mean age of participants was 46.3 years (SD = 10.2), with doctors being older (mean = 52.1; SD = 7.6) than office workers (mean = 47.3; SD = 9.3) and nurses (mean = 40.8; SD = 10.2) (*P* <0.001). The highest proportion of participants (55.6%) reported daily contact with three or fewer immigrant patients. Office workers reported more frequent contact with eight or more immigrant patients per day (28.7%), compared with nurses (9.6%) and doctors (3.3%) (*P* <0.001).

**Table 1:** Demographic characteristics and frequency of contact with immigrant patients, by health workers’ group

	**Total n = 320**	**Office workers n = 115**	**Nurses n = 114**	**Doctors n = 91**	***P* value**
	**n (%)**	**n (%)**	**n (%)**	**n (%)**	
Sex					
Female	269 (84.1)	108 (93.9)	105 (92.1)	56 (61.5)	<0.001
Male	51 (15.9)	7 (6.1)	9 (7.9)	35 (38.5)	
Number of immigrant patients daily contacted					
3 or less	178 (55.6)	51 (44.3)	59 (51.8)	68 (74.7)	<0.001
4 to 7	95 (29.7)	31 (27.0)	44 (38.6)	20 (22.0)	
8 or more	47 (14.7)	33 (28.7)	11 (9.6)	3 (3.3)	
	Mean (SD)	Mean (SD)	Mean (SD)	Mean (SD)	
Age	46.3 (10.2)	47.3 (9.3)	40.8 (10.2)	52.1 (7.6)	<0.001

### Knowledge and competencies to work with immigrant patients

Most participants (64.2%) evaluated their knowledge and competencies to work with immigrants as moderate, but 15.2% perceived them to be low. Approximately 83% of the participants considered that receiving specific training about immigrants’ health was important for improving professional performance: a higher proportion of office workers (92.0%) and nurses (85.0%) compared with doctors (68.9%) (*P* <0.001).

### Attitudes toward immigrant patients

Table 2 shows the responses of participants to the set of statements about immigrant patients. Approximately 61% disagreed that immigrant patients tend to behave like victims when they go to a health service, but 49.4% considered that some immigrants are aggressive and dangerous. Significant differences were found across workers’ groups.

**Table 2:** Responses to the statements about immigrant patients, by health workers’ group

	**Total**	**Office workers**	**Nurses**	**Doctors**	**P value**
	**n (%)**	**n (%)**	**n (%)**	**n (%)**	
Some immigrants are aggressive and dangerous					
Disagree	64 (20.0)	17 (14.8)	31 (27.2)	16 (17.6)	0.013
Do not agree nor disagree	98 (30.6)	30 (26.1)	41 (36.0)	27 (29.7)	
Agree	158 (49.4)	68 (59.1)	42 (36.8)	48 (52.7)	
Immigrants take advantage of the social benefits that Portugal offers them					
Disagree	102 (31.9)	27 (23.5)	38 (33.3)	37 (40.6)	0.001
Do not agree nor disagree	111 (34.7)	32 (27.8)	46 (40.4)	33 (36.3)	
Agree	107 (33.4)	56 (48.7)	30 (26.3)	21 (23.1)	
Immigrant patients are more claiming than the general population					
Disagree	119 (37.2)	35 (30.4)	50 (43.9)	34 (37.3)	<0.001
Do not agree nor disagree	90 (28.1)	23 (20.0)	39 (34.2)	28 (30.8)	
Agree	111 (34.7)	57 (49.6)	25 (21.9)	29 (31.9)	
Immigrants often dramatise and exaggerate their problems					
Disagree	116 (36.2)	28 (24.3)	56 (49.1)	32 (35.2)	<0.001
Do not agree nor disagree	102 (31.9)	33 (28.7)	37 (32.5)	32 (35.2)	
Agree	102 (31.9)	54 (47.0)	21 (18.4)	27 (29.6)	
Frequently immigrants do not respect the health services’ rules					
Disagree	128 (40.0)	31 (27.0)	53 (46.5)	44 (48.3)	0.001
Do not agree nor disagree	80 (25.0)	28 (24.3)	33 (28.9)	19 (20.9)	
Agree	112 (35.0)	56 (48.7)	28 (24.6)	28 (30.8)	
When immigrants come to my service they behave like victims					
Disagree	194 (60.6)	55 (47.8)	69 (60.5)	70 (76.9)	<0.001
Do not agree nor disagree	67 (21.0)	26 (22.6)	28 (24.6)	13 (14.3)	
Agree	59 (18.4)	34 (29.6)	17 (14.9)	8.8	

The multiple linear regression analysis allowed us to identify several variables associated with the dependent variable ‘attitudes toward immigrant patients’ (Table 3). Doctors (beta = 0.895; *P* = 0.004) and nurses (beta = 0.311; *P* <0.001) were more likely to show positive attitudes when compared with office workers. Among doctors, age was significantly associated with attitudes: the older ones had a lower probability of reporting positive attitudes than the younger ones (beta = −0.678; *P* = 0.030). Workers who have daily contact with three or fewer immigrant patients (beta = 0.258; P = 0.001) and with four to seven (beta = 0.209; *P* = 0.008) were more likely to report positive attitudes, compared with those workers who had contact with eight or more immigrant patients per day.

**Table 3:** **Multiple linear regression results of the dependent variable** ‘**Attitudes toward immigrant patients’**

	**Beta**	**95% CI**	**P value**
Sex^a^			
Female	- 0.034	(− 1.673-0.895)	0.55
Health workers’ group^b^			
Doctors	0.895	(2.651-13.843)	0.004
Nurses	0.311	(1.668-3.734)	<0.001
Age			
Age x Doctors	- 0.678	(−0.225- -0.012)	0.03
Number of immigrant patients daily contacted^c^			
3 or less	0.258	(0.839-3.484)	0.001
4 to 7	0.209	(0.502-3.300)	0.008
R^2^ = 0.16			

^a^ Reference group: Male sex.

^b^ Reference group: Office workers.

^c^ Reference group: Eight or more immigrant patients daily contacted.

## Discussion

The findings of this study reveal positive attitudes toward immigrant patients among health workers. This may partially result from national efforts that have been undertaken to promote integration of the immigrant population, such as the creation of the High Commission for Immigration and Intercultural Dialogue (ACIDI) in 2002. This public entity has collaborated in the development, implementation and evaluation of inclusive policies and measures [21]. More specifically, strategies have been developed to promote immigrants’ access and utilization of health services with a view to contributing to reduce health inequalities. Indeed, the current legislation in Portugal establishes the entitlement of immigrants to access to health services and their right to receive quality health care [3].

Results indicate variation in health workers’ attitudes toward immigrant patients. Several participants possibly hesitated in answering questions on these issues as immigration policy and prevention of discrimination are currently major political and social concerns. This could explain the high rate of neutral responses such as ‘do not agree nor disagree’ among participants.

The study also showed a considerable proportion of health workers reporting negative attitudes toward immigrant patients, which is consistent with other studies [13,14,22]. Similarly, a national survey demonstrated that negative attitudes toward immigrants still persist among the Portuguese population [23].

In Portugal, there is a lack of published studies on attitudes of health workers toward immigrant patients. Indeed, to our knowledge, this is the first quantitative study aimed at addressing these issues. The scarce international research has put forward that attitudes are a complex phenomenon and may vary considering different immigrant groups [13,22]. In this sense, although we did not collect information on attitudes toward specific groups of immigrants, future research should explore to what extent health workers’ attitudes differ according to patients’ socio-demographic characteristics.

This study examined whether demographic characteristics and professional experience of health workers influence attitudes toward immigrant patients. No gender or age effects were observed, in line with some investigations [14,24]. However, a significant age trend was found among doctors: the older the doctors, the less positive their attitudes. Older generations tend to be less tolerant and have stronger negative feelings toward immigrants due to a higher adhesion to conservative values, while younger people tend to adhere to more tolerant social norms, showing more openness to diversity and positive attitudes toward immigration, as shown in earlier studies [23,25].

Across health workers’ groups, office workers were less likely to express positive attitudes toward immigrant patients. This may be explained by the specific functions of office workers as they are responsible for dealing with administrative issues related to patients’ registration, which leads to different contact patterns and experiences. Our findings are important since unfavourable attitudes toward immigrants among this group may have serious implications on immigrant patients’ access and utilization of services. Additionally, office workers have a lower educational level than nurses and doctors. Previous studies have shown that individuals with lower educational levels may be less tolerant and reveal negative attitudes toward immigrants [25,26].

Health workers who reported less frequent daily contact with immigrant patients showed more positive attitudes toward this group. These results contrast with the Allport’s contact theory, which suggests that interaction among distinct groups, with similar status and common goals, reduces mutual stereotypes and conflict and promotes understanding and cooperative relations [27]. More recent research has pointed out the contact theory’s lack in addressing the nature of contact among individuals with different status and its implications [28]. Indeed, rather than attenuating conflict, contact between different groups may actually increase tension [27,29]. In line with this perspective, Pitkanen and Kouki observed that negative attitudes of Finnish authorities toward immigrants are related to the interaction experiences and especially to problems that arise from their contact with this population [26]. Similarly, our results may reveal difficulties in health worker-patient interaction. In another study conducted by our team, health workers described that interaction with immigrant patients is often difficult and provision of care generates feelings of work overload, anxiety and frustration [30], as also pointed out by other authors [20]. Nevertheless, health workers’ experiences in encounters with immigrants and its influence on attitudes should be further investigated.

In Portugal, providing care to immigrants represents a recent experience for health workers. Indeed, most participants perceived to have moderate or low competencies to work with immigrants, and considered that specific training is important for performance. These may be indicators that health staff is motivated to improve competencies and therefore interventions should be supported.

Some strengths of this research are the large and representative sample of health workers from primary health care services in the Lisbon region and the response rate of 80%, higher than that found in similar investigations [13,14]. The different demographic characteristics and professional experience across the health worker groups may have influenced the results; however, they fairly represent the heterogeneity within the health workforce.

In this study there is a possibility that the social desirability effect biased participants’ responses. Nevertheless, the strict anonymity with which the questionnaire was filled out and the fact that it was self-administered may have contributed to minimising this potential bias.

## Conclusions

Although in our study health workers reveal positive attitudes, the evidence presented reinforces the need to develop strategies to prevent negative attitudes and stereotyping. Individual characteristics, professional profile and experience should be addressed to ensure effectiveness of interventions. Improving health workers’ interaction with immigrant patients may contribute to the delivery of quality health care. In this sense, it is crucial to increase health workers’ knowledge about the specificities of communities and develop competencies to deal with culturally diverse populations. Special emphasis should be given to enhancing self-awareness of attitudes toward different immigrant groups, raising consciousness about workers’ bias and the effect of stereotypes on the quality of health care, increasing knowledge about cultural health beliefs, attitudes and practices of these populations and improving communication skills. It would also be valuable that this strategy begins at an early stage during undergraduate and graduate education. More broadly, readjustment of health systems at the organizational and structural level may have subsequent benefits in responding effectively to the needs of immigrant populations. One approach could be increasing workforce diversity at all levels of the health care organization in a way that would reflect the cultural diversity of the community served.

## Competing interests

The authors declare that they have no competing interests.

## Authors’ contributions

SD conceived and designed the study, supervised its implementation, namely data collection, helped in the statistical analysis and helped to write the manuscript. AG conducted the literature review, helped to analyse data and helped to write the manuscript. HC participated in the design and implementation of the study and helped to draft the manuscript. MOM performed the statistical analysis and assisted in finalising the manuscript. All authors read and approved the final manuscript.
